# Evaluation of Leg-foot Range of Motion: Which Measurement Method Is Most Reliable?

**DOI:** 10.1055/s-0042-1749620

**Published:** 2022-06-10

**Authors:** Marco Túlio Costa, Javier Felipe Salinas Tejerina, Cesar Augusto Lima da Silva, Itallo Epaminondas de Queiroz Rêgo, Jordanna Maria Pereira Bergamasco, Noé De Marchi Neto

**Affiliations:** 1 Grupo do Pé e Tornozelo, Departamento de Ortopedia e Traumatologia, Faculdade de Ciências Médicas da Santa Casa de São Paulo, São Paulo, SP, Brasil

**Keywords:** ankle, radiography, range of motion, articular

## Abstract

**Objective** To evaluate the methods of measuring leg-foot movement in normal ankles and feet by comparing the results of clinical measurements with those of radiographic measurement and to determine the range of leg-foot movement considered normal.

**Methods** Leg-foot movement was measured in 44 patients (60 feet) using a traditional goniometer, digital goniometer, inclinometer, *smartphone application* , in addition to radiographic measurement (considered gold standard). Maximum dorsiflexion was achieved by asking the patient to take a step forward with the contralateral foot and perform as much dorsiflexion as possible in the ankle studied without removing the heel from the ground. For maximum plantar flexion, the patient was asked to take a step back with the contralateral foot and make as much plantar flexion as possible without removing the studied forefoot from the ground.

**Results** The values obtained in radiographic measurement were higher than those obtained with clinical measurement. When we compared only the results of clinical measurement, the traditional goniometer was inaccurate. According to the radiographic method, the mean leg-foot range of motion was 65.6 degrees. The mean maximum plantar flexion was 34.9 degrees, and the mean maximum dorsiflexion was 30.7 degrees.

**Conclusions** The most appropriate method for the evaluation of leg-foot range of motion is the radiographic one. The traditional goniometer proved to be the most imprecise clinical method. The mean leg-foot range of motion in healthy young adults was 65 degrees.

## Introduction

The measurement of the range of motion of the ankle is now considered of great importance for diagnosis, therapeutic choice, and follow-up of treatment evolution in patients with pathologies, both in the ankle and the hindfoot. 1 However, there is no established method of choice for the safe assessment of talocrural mobility. 1 2 3 Some authors have published several possibilities for evaluating this movement, using from traditional goniometers, 4 5 6 digital goniometers, 3 inclinometers, 5 6 applications developed for smartphones, 4 equipment developed specifically for this purpose, 5 6 and measurement using radiographs. 7 8 Moreover, the movement between the leg and the foot does not occur alone in the ankle, but in conjunction with the movement of the other joints of the hindfoot and even the middle and forefoot 1 8 (leg-foot movement). According to Thornton et al., 3 the measurement of this leg-foot movement is more important than the measurement of ankle movement isolated, because it is this movement (leg-foot) that the patient perceives and uses on a day-to-day life. Therefore, in the evaluation of the result of some treatments or surgical procedure, it is the measurement of this movement that should be used.

With the emergence of arthroplasty for the treatment of ankle arthrosis, the measurement of the range of motion of this joint gained greater importance, as this would be one of the theoretical advantages of arthroplasty over arthrodesis. 7 8 9 As there is no safe method for measuring ankle mobility, the movement between leg and foot began to be used in this evaluation. 3 7 9 10 Hordyk et al. 8 described a method for the evaluation of ankle arthroplasties, also used by Lisboa Neto et al., 11 using radiographic examination in the incidence in profile with load, a film with the patient performing maximum plantar flexion, another film with maximum dorsiflexion. The range of motion is determined by measuring the angle between the plantar surface (ground) and the posterior cortical of the tibia. 8 Lisboa Neto et al. 11 used the longitudinal axis of the distal tibia. 11

The aim of the present study is to evaluate the methods of measuring leg-foot movement in normal ankles and feet by comparing the results of clinical measurements (traditional goniometer, digital goniometer, inclinometer, smartphone inclinometer application) with those of radiographic measurement to define the best method to be used in daily practice and determine which leg-foot range is considered normal.

## Methods

This study was carried out in the department of orthopedics and traumatology of our institution and was approved by the ethics committee on research in human beings. All participants signed the informed consent form (TCLE). The sample consisted of 44 patients aged 18 years or older, totaling 60 feet, attending the outpatient clinic of this institution. Those with changes in the hip and/or knee joints, movement restriction in both feet, amputation of one of the limbs, and previous pathologies in the lower limb to be evaluated were excluded. Adopting a statistical confidence of 95%, the sample with 60 cases has a power of 0.973 in detecting differences, which we consider satisfactory for our study.

Participants were submitted to leg-foot movement measurement with: (1) traditional goniometer; (2) digital goniometer (Digital Angle Ruler 200MM/ Shahe); (3) inclinometer (Digital Inclinometer/Digital Level); (4) smartphone application (Ratefast Goniometer v. 1.3/Alchemy Logic Systems, INC) available for IOS and Android platforms; and (5) profile radiography with maximum flexion and extension of the ankle and foot. We considered the radiographic method as the gold standard in our study. All measurements were evaluated with maximum load, plantar flexion, and dorsiflexion without removing the foot from the ground. The four clinical methods (traditional goniometer - TG, digital goniometer - DG, inclinometer – In, and application - App) were compared with radiographic measurement to verify if there was a significant difference between them.

**Fig. 1:**
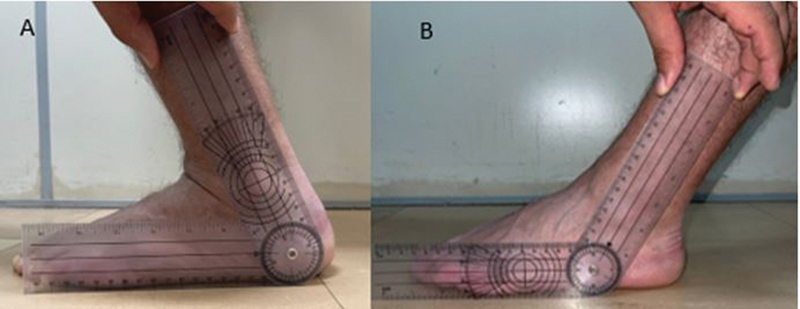
Profile foot photographs using the traditional goniometer. We used as parameter the soil and the fibula dyaphysis, **(A)** maximum dorsiflexion and **(B)** maximum plantar flexion.

**Fig. 2:**
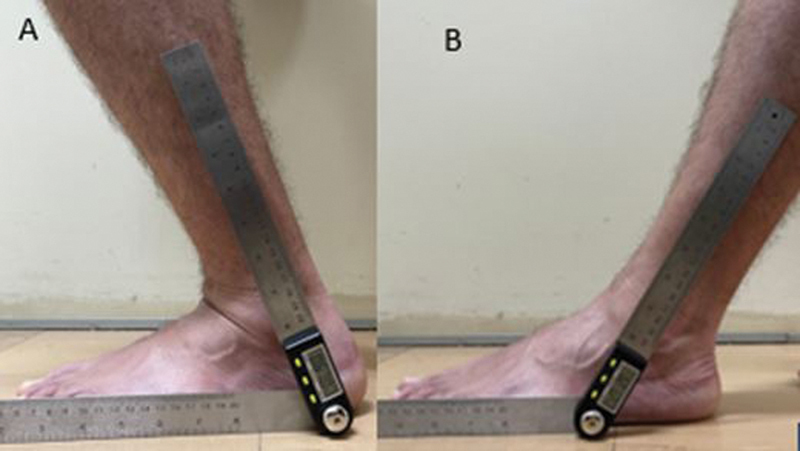
Profile foot photographs using the digital goniometer. We used as parameter the soil and the fibula dyaphysis, **(A)** maximum dorsiflexion and **(B)** maximum plantar flexion.

**Fig. 3:**
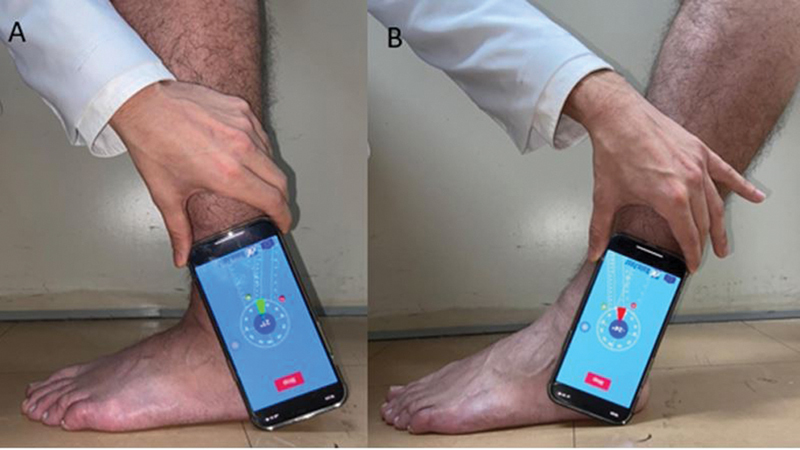
Photographs of the foot in profile using the smartphone application - aligned with the axis perpendicular to the ground and the axis of the fibula. **(A)** maximum dorsiflexion and **(B)** maximum plantar flexion.

**Fig. 4:**
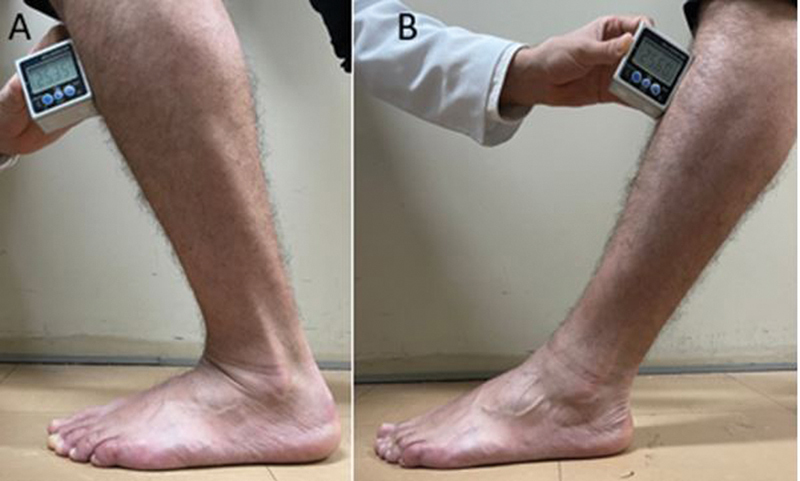
Photographs of the foot in profile using the inclinometer - positioned just below the anterior tuberosity of the tibia. **(A)** maximum dorsiflexion and **(B)** maximum plantar flexion.

**Fig. 5:**
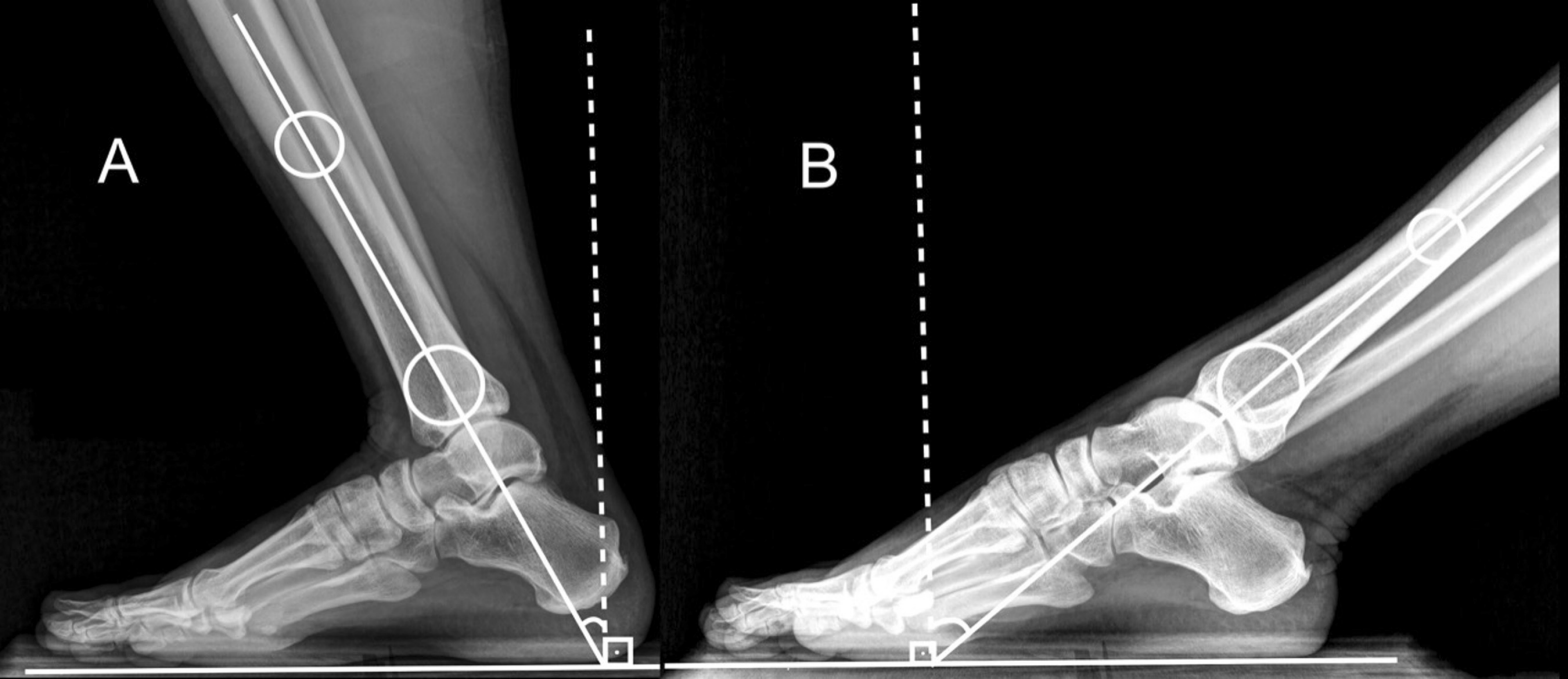
Radiographs of the foot in profile and with load demonstrating the method of measurement of joint amplitude. The longitudinal axis of the tibia and a line representing the soil were marked. **(A)** maximum dorsiflexion and **(B)** maximum plantar flexion. Source: Allinger TL, Engsberg JR. A method to determine the range of motion of the ankle joint complex, in vivo. J Biomech. 1993;26(1):69–76.

Two researchers evaluated the leg-foot movement three times in each method. The mean of the obtained values was considered as final measure. For the measurement with the traditional ( Fig. 1 ) and digital ( Fig. 2 ) goniometers, as well as with the smartphone application ( Fig. 3 ), the ground and the axis of the fibula diaphysis were used as parameters. For the inclinometer, the landmark was used immediately below the anterior tuberosity of the tibia ( Fig. 4 ). On radiographic examination, the angle between a line perpendicular to the ground axis and the central axis of the distal tibia was considered to calculate the dorsiflexion and plantar flexion of leg-foot movement ( Fig. 5 ). Maximum dorsiflexion was achieved by asking the patient to take a step forward with the contralateral foot and perform as much dorsiflexion as possible in the ankle studied without removing the heel from the ground. For maximum plantar flexion, the patient was asked to take a step back with the contralateral foot and make as much plantar flexion as possible without removing the studied forefoot from the ground ^l9,^ 11 ( Fig. 1 ).

For statistical analysis, the following software were used: IBM SPSS Statistics for Windows Version 20.0 (IBM Corp., Armonk, NY, USA), Minitab 16 and Microsoft Excel 2010 (Microsoft Corp., Redmond, WA, USA). We defined for this study a significance level of 0.05 (5%). We used the Wilcoxon test to compare the results of the range of motion obtained by the different measurement methods.

## Results

We evaluated 60 feet (44 individuals), 29 right feet and 31 left feet, 36 male and 8 female participants, with a mean age of 36 years old (ranging from 19 to 59 years).

Considering the radiographic measurement as the gold standard, we obtained an average range of motion between the leg and foot of 65.6 degrees, with a mean plantar flexion of 34.9 degrees and dorsiflexion of 30.7 degrees. Table 1 shows the data on range of motion, maximum plantar flexion, and maximum dorsiflexion obtained with clinical measurement methods. We can observe that in clinical measurements, the values obtained were lower than those noted in radiographic measurement. After statistical analysis of these data, we can state that the range of motion of the four clinical methods was different and lower than the value obtained with radiographic measurement.

**Table 1:** Results of the mean and median of the total range of leg-foot movement, maximum plantar flexion, and maximum dorsiflexion on radiography (x-ray), using the traditional goniometer (traditional), the digital goniometer (digital), the inclinometer, and the smartphone application (APP)

		Average	Median	*P* -value
Total Amplitude	RX	65.6	67	- x -
	Traditional	61.3	61	< 0.001
	Digital	63.2	64	0.001
	Inclinometer	63.3	64	0.008
	APP	62.7	63	0.002
Inflection	RX	34.9	37	- x -
	Traditional	32.7	32	< 0.001
	Digital	33.4	34	0.036
	Inclinometer	33.4	34	0.076
	APP	32.9	33.5	0.019
Extension	RX	30.7	30.5	- x -
	Traditional	28.5	28	< 0.001
	Digital	29.5	30	0.040
	Inclinometer	29.6	30	0.103
	APP	29.6	30	0.067

*P* -value was measured using the Wilcoxon method

When comparing range of motion with only clinical measurement methods (manual goniometer, digital goniometer, inclinometer, and smartphone app) we noticed that the results with the manual goniometer were statistically different from the other clinical methods. The measurements of range of motion obtained with the digital goniometer, smartphone app, and inclinometer were statistically similar.

## Discussion

Although ankle amplitude is considered important both in the diagnosis and in the follow-up of the treatment of various pathologies involving this region, there is no standardization in the literature on which method of measurement is best.

The evaluation of isolated talocrural joint is also a matter of controversy. Coetzee and Castro 7 described a method of radiographic measurement of the range of motion of the talocrural joint. Although radiography with dorsiflexion was performed with load, radiography to measure plantar flexion was performed without load, which in our opinion is a bias in the measurement of range of motion. Russell et al. 12 found differences in the range of motion in the ankle of ballerinas when measured with and without load. Hordyk et al. 8 measured leg-foot mobility and isolated talocrural joint using radiographs in profile incidence with load. However, unlike our study, in which we used the longitudinal axis of the tibia, these authors considered the posterior cortical of the tibia as tibial axis. This choice was justified, according to the authors, because this parameter is rarely obstructed on radiography, even in those cases of ankle arthroplasty with intramedullary nail. In addition to measuring the leg-foot axis, they also measured the range of motion of the ankle and for this, they traced another axis, considering the lower joint surface of the talus head and the later point of the posterior facet of the talocalcaneal articular surface, which, in our opinion, can also lead to measurement errors due to ankle and foot positioning at the time of radiography and anatomical variations. Another detail to be considered is that the tibia's posterior cortical may present alterations resulting from fractures or deformities, which would hinder the exact design of this axis. Therefore, the axis of the distal tibia as used in our study would be a measured option with lower probability of errors.

We noticed that the results of ankle range of motion using the radiographic method (65.6 degrees on average) were higher than those obtained with the other methods and with a statistically significant difference with all clinical measurements studied here. Thus, we recommend that this should be the measurement considered in clinical practice to assess the leg-foot range of motion, and we standardized in our service this radiographic evaluation for patients with ankle and foot pathologies. We could not find a clear reason why the radiographic measurement was different from the clinical measures. Perhaps, because radiography is a complementary examination, patients may have worked harder at the extremes of the movement, achieving greater range of motion.

When we evaluated the clinical methods, we noticed that they were equivalent, except for the measurement performed with the traditional goniometer, usually an orthopedist's pocket instrument. We believe that in addition to the difficulty of reading the result obtained, the short arms of this instrument and the difficulty to achieve correct positioning may have interfered in the measurement. Although some authors report similarity between clinical measurements in the literature, 13 in our study, measurement with the traditional goniometer was inaccurate to determine the true amplitude of the leg-foot movement. Marcano-Fernandez et al. 14 commented that the traditional goniometer, although widely used in orthopedics, leads to many measurement errors, and has low reliability. Russell et al., 12 evaluating ankle mobility in dancers, found different results comparing the traditional goniometer and the inclinometer. Thornton et al. 3 used a digital goniometer to measure leg-foot movement. They found different values from ours, with leg-foot range of motion of 79.8 degrees. In our study, the range of this movement measured on radiography was 65.6 degrees, a difference of 14.2 degrees. If we consider the measurement of the digital goniometer employed by us, the difference was even greater. The measurement in our study was 63.2 degrees, and the difference in the result of the two studies was 16.4 degrees. Thornton et al. 3 used a goniometer with 50 cm arms and 20 cm knots. Considering the longitudinal axis of the fibula as a parameter, it may be that the longer arm goniometer results in a more precise measurement. However, this is not the biggest difference between the two studies. Thornton et al. 3 measured plantar flexion with the patient sitting in a chair, thus eliminating the effect of weight discharge on the measure, a fact that we considered important. Although it allows a more comfortable position to the patient in plantar flexion measurement, the effect of weight discharge can change the functioning of these joints. In addition, we must remember that these joints work on a day-to-day high with weight discharge. Therefore, we believe that for a more reliable measure, these measurements should be carried out with load. When we evaluated the isolated dorsiflexion movement, the value of 29.6 degrees obtained by Thornton et al. 3 was similar to that obtained in the radiographic study, 30.7 degrees. The great difference was observed in the plantar flexion values, 51.2 degrees in Thornton's study and 34.9 degrees in our study. Grimston et al. 15 observed differences in range of motion that they called the ankle joint complex, according to the age and gender of the patients evaluated. In our study, the mean age of the patients was 36 years old. According to the study by Grimston et al., 15 who employed an equipment developed to measure the movement of the ankle complex (ankle joint and talocalcaneal), the mean range of motion of the ankle joint complex in this age group would be 74.2 degrees, ranging from 57 degrees to 92 degrees. Also different from the values we obtained in our study (65.6 degrees).

The goniometer is a low-cost instrument widely used in clinical practice. The traditional, with shorter rods, requires greater training and attention from the evaluator both for the correct positioning of the rotation fulcrum and alignment of the arms of the instrument with the correct axes and reference points. The digital version that we used in this study has longer arms (20 cm), which facilitates better alignment with the leg and foot axes and allows the measurement to be more accurate. Thus, it is not necessary to adjust the visual field of the examiner to write down the exact measurement. Although the digital inclinometer was used in some articles 5 6 as a measure of the ankle range of motion and its measurement is statistically similar to the other clinical methods used in this study, except the traditional goniometer, in our opinion, its correct use is technically more difficult. Its correct positioning with the tibial tuberosity is not always easy and requires care and attention on the part of the examiner, which can lead to measurement errors. For this reason, we do not recommend this method in the day-to-day of the office, due to greater technical difficulty to obtain the results. Smartphone apps are available and can assist in the measurement of range of motion. Wang et al. 4 compared three applications available with the traditional goniometer and did not find statistically significant differences. However, our results discourage the use of the traditional goniometer as a benchmark. The application used in this study did not show statistical difference with the other clinical methods, but there was a significant difference from the result obtained on radiography, and, for this reason, we did not use it routinely.

This study has limitations. Clinical measurements were performed simultaneously in all patients. There was no pretest, simulating and mesuring the movement, so that patients could become familiar with the method. The radiographic measurement was performed at a different time, after clinical measurement, and there was no randomization or draw of the sequence of the methods employed, which can also be considered a bias of the work. However, clinical measurements made at the same time, guarantee a better fidelity to the result. It was not possible to perform clinical measurements in the radiographic examination room, because the delay could cause delays in the care of patients who require the examination. The researchers were not blinded to the results of the clinical measures applied just before, which can also be considered a bias.

Despite the several articles that employ clinical measurement of ankle range of motion, we believe, based on the results of this study, that the best evaluation method is the radiographic one. Therefore, we introduced the radiographic examination in profile with maximum plantar flexion and maximum dorsiflexion routinely at our outpatient clinic. We also believe that the isolated measurement of the movement of the talocrural joint is difficult to evaluate in daily clinical practice and recommend the use of leg-foot range of motion.

## Conclusions

The most appropriate method for the evaluation of leg-foot range of motion is radiographic. The traditional goniometer proved to be the most imprecise clinical method in this study. The mean leg-foot range of motion in healthy young adults was 65 degrees.
