# Supplementary material for: Evaluation of Leg-foot Range of Motion: Which Measurement Method Is Most Reliable?
Source: Rev Bras Ortop (Sao Paulo). 2022 Jun 10;59(6):e907–12. doi: 10.1055/s-0042-1749620 (PMC11663077; doi:10.1055/s-0042-1749620)
Supplement: Related texts

# Research article: Avaliação da amplitude do movimento perna-pé: Qual método de aferição é mais fidedigno?

**Keywords:** amplitude de movimento articular, radiografia, tornozelo

## Abstract

**Objetivo** Avaliar os métodos de mensuração do movimento perna-pé em tornozelos e pés normais comparando os resultados das medidas clínicas com a mensuração radiográfica e determinar qual é a amplitude do movimento perna-pé considerada normal.

**Métodos** O movimento perna-pé foi mensurado em 44 pacientes (60 pés) empregando um goniômetro tradicional, goniômetro digital, inclinômetro, aplicativo para *smartphone* , além da mensuração radiográfica (considerada padrão ouro). A dorsiflexão máxima foi alcançada pedindo ao paciente dar um passo à frente com o pé contralateral e realizar o máximo de dorsiflexão possível no tornozelo estudado sem retirar o calcanhar do solo. Já para a flexão plantar máxima, foi solicitado ao paciente para dar um passo para trás com pé contralateral e fazer o máximo de flexão plantar possível sem retirar o antepé estudado do solo.

**Resultados** Os valores obtidos na mensuração radiográfica foram maiores do que os obtidos com a mensuração clínica. Quando comparamos apenas os resultados da mensuração clínica, o goniômetro tradicional se mostrou impreciso. Segundo o método radiográfico, a média de amplitude do movimento perna-pé foi de 65,6 graus. Já a média da flexão plantar máxima foi de 34,9 graus, e a média da dorsiflexão máxima foi de 30,7 graus.

**Conclusões** O método mais adequado para a avaliação da amplitude do movimento perna-pé é o radiográfico. O goniômetro tradicional se mostrou o método clínico mais impreciso. A média de amplitude do movimento perna-pé em adultos jovens e saudáveis foi de 65 graus.

## Introdução

A mensuração da amplitude de movimento do tornozelo é hoje considerada de grande importância para o diagnóstico, escolha terapêutica e acompanhamento da evolução do tratamento nos pacientes com patologias, tanto do tornozelo como de todo retropé. 1 No entanto, não há um método de escolha estabelecido para a avaliação segura da mobilidade talocrural. 1 2 3 Alguns autores publicaram diversas possibilidades de avaliação deste movimento, empregando desde goniômetros tradicionais, 4 5 6 goniômetros digitais, 3 inclinômetros, 5 6 aplicativos desenvolvidos para *smartphones* , 4 equipamentos desenvolvidos especificamente para este fim 5 6 e mensuração empregando radiografias. 7 8 Além do mais, o movimento entre a perna e o pé não ocorre isoladamente no tornozelo, e sim em conjunto com o movimento das outras articulações do retropé e até do médio e antepé 1 8 (movimento perna-pé). Segundo Thornton et al. 3 , a mensuração deste movimento perna-pé é mais importante que a medida do movimento do tornozelo isolado, porque é este movimento (perna-pé) que o paciente percebe e utiliza no dia a dia. Portanto, na avaliação do resultado de algum tratamento ou procedimento cirúrgico, é a mensuração deste o movimento que deveria ser empregada.

Com o surgimento da artroplastia para o tratamento da artrose do tornozelo, a mensuração da amplitude de movimento desta articulação ganhou maior importância, pois esta seria uma das vantagens teóricas da artroplastia sobre a artrodese. 7 8 9 Como não há método seguro para a mensuração da mobilidade do tornozelo, o movimento entre a perna e o pé começou a ser utilizado nesta avaliação. 3 7 9 10 Hordyk et al. 8 descreveram um método para a avaliação das artroplastias do tornozelo, também empregado por Lisboa Neto et al. 11 , utilizando exame radiográfico na incidência em perfil com carga, um filme com o paciente realizando flexão plantar máxima, outro filme com dorsiflexão máxima. A amplitude de movimento é dada pela mensuração do ângulo entre a superfície plantar (solo) e a cortical posterior da tíbia. 8 Lisboa Neto et al. 11 utilizaram o eixo longitudinal da tíbia distal. 11

O objetivo deste estudo é avaliar os métodos de mensuração do movimento perna-pé em tornozelos e pés normais e comparar os resultados das medidas clínicas (goniômetro tradicional, goniômetro digital, inclinômetro, aplicativo inclinômetro para *smartphone* ) com a mensuração radiográfica, com intuito de definir qual o melhor método para ser empregado na prática diária e determinar qual é a amplitude do movimento perna-pé considerada normal.

## Métodos

Este trabalho foi realizado no departamento de ortopedia e traumatologia da nossa instituição e foi aprovado no comitê de ética em pesquisa em seres humanos. Todos os participantes assinaram o termo de consentimento livre e esclarecido (TCLE). A amostra foi composta de 44 pacientes, total de 60 pés, frequentadores do ambulatório dessa instituição, com idade maior ou igual a 18 anos. Foram excluídos aqueles com alterações nas articulações do quadril e/ou joelho, restrição de movimento em ambos os pés, amputação de um dos membros, e patologias prévias no membro inferior a ser avaliado. Adotando uma confiança estatística de 95%, a amostra com 60 casos tem um poder de 0,973 em detectar diferenças, que consideramos satisfatório para nosso estudo.

Os participantes foram submetidos à mensuração do movimento perna-pé com: (1) goniômetro tradicional; (2) goniômetro digital (Digital Angle Ruler 200MM/ Shahe); (3) inclinômetro (Digital Inclinometer/Digital Level); (4) aplicativo para *smartphone* (Ratefast Goniometer v. 1.3/Alchemy Logic Systems, INC) disponível para as plataformas IOS e Android; e com (5) radiografia em perfil com flexão e extensão máxima do tornozelo e pé. Consideramos o método radiográfico como padrão ouro no nosso estudo. Todas as medidas foram avaliadas com carga, em flexão plantar e dorsiflexão máximas sem retirar a planta do pé do solo. Os quatro métodos clínicos (goniômetro tradicional - GT, goniômetro digital - GD, inclinômetro – In, e aplicativo - App) foram comparados com a mensuração radiográfica para verificar se existe diferença significativa entre eles.

Dois pesquisadores avaliaram o movimento perna-pé três vezes em cada método. A média dos valores obtidos foi considerada como medida final. Para a medida com os goniômetros tradicional ( Fig. 1 ) e digital ( Fig. 2 ), assim como no aplicativo do *smartphone* ( Fig. 3 ), foram utilizados como parâmetros o solo e o eixo da diáfise da fíbula. Para o inclinômetro, utilizou-se o marco imediatamente abaixo da tuberosidade anterior da tíbia ( Fig. 4 ). No exame radiográfico, foi considerado o ângulo entre uma linha perpendicular ao eixo do solo e o eixo central da tíbia distal para calcular a dorsiflexão e flexão plantar do movimento perna-pé ( Fig. 5 ). A dorsiflexão máxima foi alcançada pedindo ao paciente dar um passo à frente com o pé contralateral e realizar o máximo de dorsiflexão possível no tornozelo estudado sem retirar o calcanhar do solo. Já para a flexão plantar máxima, foi solicitado ao paciente para dar um passo para trás com pé contralateral e fazer o máximo de flexão plantar possível sem retirar o antepé estudado do solo 9 11 ( Fig. 1 ).

**Fig. 1:** Fotografias do pé em perfil utilizando o Goniômetro tradicional. Utilizamos como parâmetro o solo e a diáfise da fíbula, **(A)** dorsiflexão máxima e **(B)** flexão plantar máxima.

**Fig. 2:** Fotografias do pé em perfil utilizando o goniômetro digital. Utilizamos como parâmetro o solo e a diáfise da fíbula, **(A)** dorsiflexão máxima e **(B)** flexão plantar máxima.

**Fig. 3:** Fotografias do pé em perfil utilizando o aplicativo de smartphone - alinhado com o eixo perpendicular ao solo e ao eixo da fíbula. **(A)** dorsiflexão máxima e **(B)** flexão plantar máxima.

**Fig. 4:** Fotografias do pé em perfil utilizando o inclino#x00F4;metro – posicionado logo abaixo da tuberosidade anterior da tíbia. **(A)** dorsiflexão máxima e **(B)** flexão plantar máxima.

**Fig. 5:** Radiografias do pé em perfil e com carga demonstrando o método de aferição de amplitude articular. Foram marcados o eixo longitudinal da tíbia e uma linha que representa o solo. **(A)** dorsiflexão máxima e **(B)** flexão plantar máxima. Fonte: Allinger TL, Engsberg JR. A method to determine the range of motion of the ankle joint complex, in vivo. J Biomech. 1993;26(1):69–76.

Para análise estatística foram utilizados os softwares: IBM SPSS Statistics for Windows Versão 20.0 (IBM Corp., Armonk, NY, EUA), Minitab 16 (Minitab LLC, Stste College, PA, EUA), e Microsoft Excel Office (Microsoft Corp., Redmond, WA, EUA). Definimos para este trabalho um nível de significância de 0,05 (5%). Empregamos o teste de Wilcoxon para comparar os resultados da amplitude de movimento obtidos pelos diferentes métodos de mensuração.

## Resultados

Foram avaliados 60 pés (44 indivíduos), 29 pés direitos e 31 esquerdos, sendo 36 participantes do sexo masculino e 8 do sexo feminino, com média de idade de 36 anos (variando de 19–59 anos).

Considerando a medida radiográfica como padrão ouro, obtivemos uma média de amplitude de movimento entre a perna e o pé de 65,6 graus, sendo a média de flexão plantar de 34,9 graus e de dorsiflexão de 30,7 graus. Na Tabela 1 estão os dados da amplitude de movimento, flexão plantar máxima e dorsiflexão máxima obtidos com os métodos da mensuração clínica. Podemos observar que nas mensurações clínicas, os valores obtidos foram menores do que aqueles obtidos na mensuração radiográfica. Após análise estatística destes dados, podemos afirmar que a amplitude do movimento dos quatro métodos clínicos foi diferente e menor que o valor obtido na mensuração radiográfica.

**Tabela 1:** Resultados da média e mediana da amplitude total do movimento perna- pé, flexão plantar máxima e dorsiflexão máxima na radiografia (RX), utilizando o goniômetro tradicional (tradicional), o goniômetro digital (digital), o inclinômetro e o aplicativo do smartphone (APP)

		Média	Mediana	Valor de *p*
Amplitude Total	RX	65,6	67	- x -
	Tradicional	61,3	61	< 0,001
	Digital	63,2	64	0,001
	Inclinômetro	63,3	64	0,008
	APP	62,7	63	0,002
Flexão	RX	34,9	37	- x -
	Tradicional	32,7	32	< 0,001
	Digital	33,4	34	0,036
	Inclinômetro	33,4	34	0,076
	APP	32,9	33,5	0,019
Extensão	RX	30,7	30,5	- x -
	Tradicional	28,5	28	< 0,001
	Digital	29,5	30	0,040
	Inclinômetro	29,6	30	0,103
	APP	29,6	30	0,067

O valor de *p* foi medido pelo método de Wilcoxon

Quando comparamos a amplitude de movimento obtida somente usando os métodos de mensuração clínica (goniômetro manual, goniômetro digital, inclinômetro e APP do smartphone), notamos que os resultados com o goniômetro manual foram estatisticamente diferentes dos outros métodos clínicos. As medidas da amplitude do movimento aferidas com o goniômetro digital, APP do *smartphone* e inclinômetro foram estatisticamente semelhantes.

## Discussão

Apesar da amplitude do tornozelo ser considerada importante tanto no diagnóstico como no acompanhamento do tratamento de diversas patologias que envolvem esta região, não há na literatura uma padronização sobre qual o melhor método de aferição.

A avaliação da articulação talocrural isolada também é motivo de controvérsia. Coetzee e Castro 7 descreveram um método de mensuração radiográfica da amplitude de movimento da articulação talocrural. Apesar da radiografia com dorsiflexão ter sido realizada com carga, a radiografia para mensuração da flexão plantar, foi realizada sem carga, o que na nossa opinião é um viés na mensuração da amplitude de movimento. Russel et al. 12 encontraram diferenças na amplitude de movimento no tornozelo de bailarinas quando mensurados com e sem carga. Hordyk et al., 8 mensuraram a mobilidade perna-pé e da articulação talocrural isolada, utilizando radiografias na incidência em perfil com carga. No entanto, diferente do nosso estudo, no qual empregamos o eixo longitudinal da tíbia, estes autores consideraram a cortical posterior da tíbia como eixo tibial. Esta escolha foi justificada, segundo os autores, por este parâmetro raramente estar obstruído na radiografia, mesmo naqueles casos de artroplastia do tornozelo com hastes intramedulares. Além de medir o eixo perna-pé, também mediram a amplitude de movimento do tornozelo e para isto, traçaram um outro eixo, considerando a superfície articular inferior da cabeça do tálus e o ponto mais posterior da faceta posterior da superfície articular talocalcânea, o que na nossa opinião também pode levar a erros de medida, devido ao posicionamento do tornozelo e do pé no momento da radiografia e variações anatômicas. Outro detalhe a ser considerado é que a cortical posterior da tíbia pode apresentar alterações decorrentes de fraturas ou deformidades, que dificultariam o desenho exato deste eixo. Portanto, o eixo da tíbia distal como utilizado no nosso estudo, seria uma opção medida com menor probabilidade de erros.

Notamos que os resultados da amplitude de movimento do tornozelo empregando o método radiográfico (65,6 graus em média) foram maiores do que as obtidas com os outros métodos e com diferença estatisticamente significante com todas as mensurações clínicas estudadas aqui. Assim, recomendamos que esta deva ser a mensuração considerada na prática clínica para avaliação da amplitude do movimento perna-pé e padronizamos no nosso serviço, esta avaliação radiográfica para os pacientes com patologias do tornozelo e pé. Não conseguimos encontrar uma razão clara para que a medida radiográfica fosse diferente das medidas clínicas. Talvez, devido a radiografia ser um exame complementar, os pacientes podem ter se esforçado mais nos extremos do movimento, conseguindo amplitude de movimento maior.

Quando avaliamos métodos clínicos, notamos que eles são equivalentes, exceto a medida realizada com o goniômetro tradicional, normalmente instrumento de bolso do ortopedista. Acreditamos que além da dificuldade de ler o resultado obtido, os braços curtos deste instrumento e a dificuldade do correto posicionamento podem ter interferido na aferição. Apesar de alguns autores relatarem semelhança entre as mensurações clínicas na literatura, 13 no nosso estudo a mensuração com o goniômetro tradicional se mostrou imprecisa para medir a verdadeira amplitude do movimento perna-pé. Marcano-Fernandez et al. 14 comentaram que o goniômetro tradicional, apesar de amplamente utilizado na ortopedia, leva a muitos erros de medida e tem baixa confiabilidade. Russel et al., 12 avaliando a mobilidade do tornozelo em bailarinas, encontraram resultados diferentes comparando o goniômetro tradicional e o inclinômetro. Thornton et al. 3 empregaram um goniômetro digital para mensuração do movimento perna-pé. Encontraram valores diferentes dos nossos, com amplitude de movimento perna-pé de 79,8 graus. No nosso estudo, a amplitude deste movimento mensurada na radiografia foi de 65,6 graus, uma diferença de 14,2 graus. Se considerarmos a medida do goniômetro digital por nós empregado, a diferença foi ainda maior. A medida no nosso estudo foi de 63,2 graus e a diferença do resultado dos dois estudos de 16,4 graus. Thornton et al. 3 utilizaram um goniômetro com braços de 50 cm e nós de 20 cm. Considerando o eixo longitudinal da fíbula como parâmetro, pode ser que o goniômetro de braço mais longo resulte em uma medida mais precisa. No entanto, esta não é a maior diferença entre os dois estudos. Thornton et al. 3 mensuraram a flexão plantar com o paciente sentado em uma cadeira, eliminando assim o efeito da descarga do peso na medida, fato que consideramos importante. Embora permita uma posição mais cômoda ao paciente na aferição da flexão plantar, o efeito da descarga de peso pode mudar o funcionamento destas articulações. Além disto, devemos lembrar que estas articulações trabalham no dia a dia com a descarga de peso. Portanto, acreditamos que para uma medida mais fidedigna, estas mensurações devem ser realizadas com carga. Quando avaliamos o movimento de dorsiflexão isolada, o valor obtido por Thornton et al. 3 de 29,6 graus é semelhante ao que obtivemos no estudo radiográfico, 30,7 graus. A grande diferença foi observada nos valores da flexão plantar, 51,2 graus no estudo de Thornton e 34,9 graus no nosso estudo. Grimston et al. 15 observaram diferenças na amplitude de movimento que chamaram de complexo articular do tornozelo, de acordo com a idade e gênero dos pacientes avaliados. No nosso estudo, a média de idade dos pacientes foi de 36 anos. Segundo o estudo de Grimston et al., 15 que empregaram um equipamento desenvolvido para mensurar o movimento do complexo do tornozelo (articulação do tornozelo e talocalcânea), a média amplitude de movimento do complexo articular do tornozelo nesta faixa etária seria de 74,2 graus, variando de 57 graus a 92 graus. Também diferente dos valores que obtivemos no nosso estudo (65,6 graus).

O goniômetro é um instrumento de baixo custo e amplamente empregado na prática clínica. O tradicional, com hastes mais curtas, exige do avaliador maior treinamento e atenção tanto para o correto posicionamento do fulcro de rotação como alinhamento dos braços do instrumento com os eixos e pontos de referência corretos. A versão digital que utilizamos neste estudo tem braços mais longos (20 cm). Isso facilita o melhor alinhamento com os eixos da perna e do pé e a medida se torna mais exata, pois não é necessário ajuste do campo visual do examinador para anotar a medida exata. Apesar de o inclinômetro digital ter sido empregado em alguns artigos 5 6 como medida da amplitude de movimento do tornozelo e de a sua medida ser estatisticamente semelhante aos outros métodos clínicos empregados neste trabalho - exceto o goniômetro tradicional - na nossa opinião, o seu correto uso é tecnicamente mais difícil. Seu correto posicionamento junto à tuberosidade tibial nem sempre é fácil e exige cuidado e atenção por parte do examinador, o que pode levar a erros de medida. Por este motivo, não recomendamos este método no dia a dia do consultório, devido a maior dificuldade técnica para obter os resultados. Aplicativos para *smartphone* estão disponíveis e podem auxiliar na aferição da amplitude de movimento. Wang et al. 4 compararam três aplicativos disponíveis com o goniômetro tradicional e não encontraram diferenças estatisticamente significantes. No entanto, os nossos resultados desencorajam o uso do goniômetro tradicional como parâmetro de comparação. O aplicativo utilizado neste estudo não demonstrou diferença estatística em relação aos outros métodos clínicos, mas houve diferença significativa em relação ao resultado obtido na radiografia, e por este motivo não o utilizamos rotineiramente.

Este estudo tem limitações. As mensurações clínicas foram realizadas simultaneamente em todos os pacientes. Não houve um pré-teste, simulando e mesurando o movimento, para que os pacientes se familiarizassem com o método. A medida radiográfica foi realizada em um momento diferente, após a mensuração clínica e não houve randomização ou sorteio da sequência dos métodos empregados, o que também pode ser considerado um viés do trabalho. No entanto, as mensurações clínicas realizadas ao mesmo tempo garantem uma melhor fidelidade ao resultado. Não foi possível realizar as medidas clínicas na sala do exame radiográfico, porque a demora poderia causar atrasos no atendimento dos pacientes que necessitam do exame. Os pesquisadores não estavam cegados aos resultados das medidas clínicas aplicadas logo antes, o que também pode ser considerado um viés.

Apesar dos diversos artigos que empregam mensuração clínica da amplitude de movimento do tornozelo, acreditamos, baseado nos resultados deste estudo, que o melhor método de avaliação seja o radiográfico. Por isto, introduzimos a realização do exame radiográfico em perfil com flexão plantar máxima e dorsiflexão máxima na rotina do nosso ambulatório. Acreditamos também que a mensuração isolada do movimento da articulação talocrural seja de difícil avaliação na prática clínica diária e recomendamos a utilização da amplitude de movimento perna-pé.

## Conclusões

O método mais adequado para a avaliação da amplitude de movimento perna-pé é o radiográfico. O goniômetro tradicional se mostrou o método clínico mais impreciso neste estudo. A média de amplitude do movimento perna-pé em adultos jovens e saudáveis foi de 65 graus.
